# Local Innate Responses to TLR Ligands in the Chicken Trachea

**DOI:** 10.3390/v8070207

**Published:** 2016-07-22

**Authors:** Neda Barjesteh, Tamiru Negash Alkie, Douglas C. Hodgins, Éva Nagy, Shayan Sharif

**Affiliations:** Department of Pathobiology, University of Guelph, Guelph, ON N1G 2W1, Canada; nbarjest@uoguelph.ca (N.B.); talkie@uoguelph.ca (T.N.A.); dhodgins@uoguelph.ca (D.C.H.); enagy@ovc.uoguelph.ca (É.N.)

**Keywords:** chicken trachea, TLR ligands, innate antiviral responses

## Abstract

The chicken upper respiratory tract is the portal of entry for respiratory pathogens, such as avian influenza virus (AIV). The presence of microorganisms is sensed by pathogen recognition receptors (such as Toll-like receptors (TLRs)) of the innate immune defenses. Innate responses are essential for subsequent induction of potent adaptive immune responses, but little information is available about innate antiviral responses of the chicken trachea. We hypothesized that TLR ligands induce innate antiviral responses in the chicken trachea. Tracheal organ cultures (TOC) were used to investigate localized innate responses to TLR ligands. Expression of candidate genes, which play a role in antiviral responses, was quantified. To confirm the antiviral responses of stimulated TOC, chicken macrophages were treated with supernatants from stimulated TOC, prior to infection with AIV. The results demonstrated that TLR ligands induced the expression of pro-inflammatory cytokines, type I interferons and interferon stimulated genes in the chicken trachea. In conclusion, TLR ligands induce functional antiviral responses in the chicken trachea, which may act against some pathogens, such as AIV.

## 1. Introduction

Avian influenza virus (AIV), which belongs to the family of *Orthomyxoviridae*, is an enveloped virus with a negative sense, single-stranded and segmented RNA [1]. With recent outbreaks of highly pathogenic avian influenza (HPAI) and low pathogenic avian influenza (LPAI) viruses, there is a need for better understanding of host responses, especially in the respiratory system to AIVs in order to develop and improve prophylactic procedures for control of AIV. Following infection of the respiratory tract with AIV, the host must produce appropriate immune responses in a short period of time to limit viral replication and eliminate the virus.

Host immune responses in the respiratory system, including innate and adaptive immune responses, are elicited by pathogens. Innate responses provide the first line of defense against influenza virus at mucosal surfaces aiming to block the entry of the virus and viral replication. However, when the virus passes the preliminary barriers, the airway epithelial cells are the primary target for the virus. Pathogenicity of AIV in chickens is initiated by the same phenomenon as mentioned above, by infecting the tracheal cells by influenza virus [2,3]. In chickens, despite the absence of essential lymphoid tissues or mucosa-associated lymphoid tissues in the trachea, the trachea is able to mount immune responses against different pathogens [4,5,6,7]. Chemoattractants produced by the trachea recruit other cells of the immune system, such as macrophages and lymphocytes, toward the trachea to limit spread of the virus [4,5,6,8]. Airway epithelial cells are of prime importance in host responses against AIV. AIV replicates in epithelial cells, and these infected epithelial cells then transport viral antigens to antigen presenting cells [9,10].

The attachment and entry of influenza virus into the host cells depend on the expression of sialic acids (SAs) and terminal sugar residues—such as galactose (Gal), *N*-acetylglucosamine (GlcNAc) or *N*-acetylgalactosamine (GalNAc)—by epithelial cells [11]. Chicken tracheal epithelium contains three types of cells (ciliated cells, goblet cells and basal cells) which express both SAα2–6Gal and SAα2–3Gal linkages [12]. However, distribution and expression of these receptors vary among different types of chicken tracheal cells [12,13]. AIV replicates in all types of cell of the tracheal epithelium, however, ciliated cells are more sensitive to AIV compared to goblet and basal cells [3,12].

Once the virus infects the host, host cells detect the presence of viral components by means of germline encoded pattern recognition receptors (PRRs). There are some differences between mammalian species and chickens in the way they sense presence of the virus via PRRs. In vertebrate species, three types of PRRs engage to recognize influenza virus: Toll-like receptors (TLRs), nucleotide oligomerization domain (NOD)-like receptors (NLRs) and retinoic acid-inducible gene-1 (RIG-1)-like receptors (RLRs) [14,15,16]. In mammals, TLR3 plays a key role in recognition of double-stranded RNA (dsRNA) once the virus replicates in host cells [14,17]. While the NLR and the RLR families recognize the presence of the virus in the cytosol of infected cells, TLR7 senses the presence of viral RNA in the endosome of host cells, such as plasmacytoid dendritic cells (pDCs) [18]. Like mammalian species, several PRRs including TLRs and RLRs have been recognized in chickens. In chickens, the expression of TLRs in trachea is up-regulated following AIV infection [19]. Despite the lack of RIG-1 in chickens to sense AIV infection, AIV is sensed intracellularly by chicken melanoma differentiation-associated protein 5 (chMDA5), which results in the expression of type I interferons (IFNs). ChMDA5 and downstream mediators of cell signaling, such as mitochondrial adaptor molecule CARDIF and chicken interferon regulatory factor 7 (chIRF7) are necessary to induce antiviral responses [20,21].

The activation of PRRs and intracellular signaling pathways result in the production of type I IFNs and pro-inflammatory responses. Type I IFNs trigger an orchestra of antiviral responses against influenza viruses including the expression of IFN-stimulated genes (ISGs) to limit the viral replication and induce adaptive immune responses [22]. In chickens, type I IFNs including chIFN-α and chIFN-β induce the expression of ISGs, such as protein kinase R (PKR) and the 2′–5′ oligoadenylate synthetase (2′,5′-OAS) [23]. Downstream ISGs, such as IFN-induced transmembrane protein (IFITM) and viperin interfere with the viral replication cycle at different stages [24,25,26].

TLR ligands have been successfully used in chickens as adjuvants or stand-alone antiviral reagents [27,28,29,30,31]. These studies have demonstrated that TLR ligands induce the expression of TLRs and antiviral responses and the production of cytokines and chemokines. TLR2, 4, and 21 ligands have been employed in vitro to treat chicken macrophages prior to AIV infection and resulting in the reduction of AIV replication in chicken macrophages [31]. The same panel of TLR ligands and the ligand for TLR7 have also been administrated in vivo in chickens to induce antiviral responses and resulting in the reduction of AIV shedding in infected chickens. These TLR ligands reduced oral and cloacal viral shedding in infected chickens. TLR21 ligand CpG ODN 1826 is the most efficacious TLR ligand to reduce oral viral shedding, while TLR4 ligand lipopolysaccharide (LPS) from *Escherichia coli* (*E.coli*) 026:B6 causes the highest reduction in cloacal viral shedding [29]. However, there is little information about induced antiviral responses in the chicken respiratory system following treatment with TLR ligands and the underlying mechanisms which are involved in the induction of antiviral responses. The purpose of this study was to determine first whether the chicken trachea responds to TLR ligands, and second whether these responses induced by TLR ligands are capable to limit AIV replication. In this study, we profiled antiviral responses in the chicken trachea following stimulation of tracheal organ culture (TOC) with TLR ligands. We also confirmed that soluble factors, secreted by chicken tracheal cells stimulated with TLR ligands, had antiviral activity against AIV.

## 2. Materials and Methods

### 2.1. Tracheal Organ Culture (TOC)

TOC was performed as previously described with some modifications [2]. Briefly, tracheas were aseptically collected from 20-day-old specific-pathogen-free (SPF) chicken embryos. Tracheas were washed twice with warm Hanks’ balanced salt solution (HBSS, Gibco, Burlington, ON, Canada) to remove excess mucus. The connective tissues surrounding the trachea were removed by careful dissection. Tracheas were dissected manually into 1 mm rings using a microtome blade. Five tracheal rings from one embryo were transferred into 24-well culture plates containing Medium 199 (Sigma-Aldrich, Oakville, ON, Canada) supplemented with 25 mM 4-(2-hydroxyethyl)-1-piperazineethanesulfonic acid (HEPES) buffer (Gibco), 200 U/mL penicillin, 80 µg/mL streptomycin, and 50 µg/mL gentamicin. Rings were incubated at 37 °C on a low speed rotator for two hours and then the medium was replaced. During the experiments, the ciliary activity of the tracheal rings was monitored.

### 2.2. Avian Influenza Virus (AIV)

A/Duck/Czech/56 (H4N6), a low pathogenic avian influenza virus (LPAIV), was used in this study. The virus was propagated in 11-day embryonated chicken eggs. Virus propagation and titration were carried out as previously described [32].

### 2.3. Stimulation of TOC with TLR Ligands

For stimulation of tracheal rings with TLR ligands, tracheal rings were cultured in 24-well plates containing five rings per well. Tracheal rings were stimulated with two concentrations of the following TLR ligands: 1.0 and 10 µg/mL Pam3CSK4 (Invivogen, San Diego, CA, USA), 1.0 and 10 µg/mL synthetic class B CpG ODN 1826 (5′-TCCATGACGTTCCTGACGTT-3′) (Sigma-Aldrich) and 1.0 and 10 µg/mL LPS derived from *E.coli* O26:B6 (Sigma-Aldrich). The control groups received 10 µg/mL synthetic non-CpG ODN (5′-TGCTGCTTGTGCTTTTGTGCTT-3′) (Sigma-Aldrich) or medium in a final volume of 500 μL. After two hours of stimulation with TLR ligands, tracheal rings were washed twice with complete medium, and then incubated at 37 °C in fresh medium. At 3, 8 and 18 h post-stimulation, tracheal rings were collected for RNA extraction. There were six biological replicates (6 individual embryos) for each treatment group.

In a subsequent experiment, to evaluate antiviral activities produced by chicken tracheas, tracheal rings were stimulated with optimum (based on results from a previous experiment) concentrations of TLR ligands. After two hours of stimulation with TLR ligands, tracheal rings were washed twice with complete medium, and then incubated at 37 °C in fresh medium. Tracheal supernatants were collected after 24 and 48 h of stimulation with TLR ligands and bioactive antiviral activity was assessed by treating cells of a chicken macrophage line (MQ-NCSU cells) with culture supernatants before infecting them with AIV.

### 2.4. cDNA Synthesis and Real-Time PCR

Total RNA was extracted from five tracheal rings using Trizol reagent (Life Technologies, Burlington, ON, Canada), according to the manufacturer’s recommendations. There were 6 biological replicates in each group and each replicate contained five rings of trachea from the same individual embryo. DNA-Free™ kit (Ambion, Austin, TX, USA) was used to remove contaminating DNA from RNA samples before cDNA synthesis. One microgram of RNA was used for cDNA synthesis. Superscript II First Strand Synthesis kit (Life Technologies) and Oligo(dT)20 primers were used for cDNA synthesis according to the manufacturer’s protocols. Quantitative real-time PCR was performed on diluted cDNA (1:10 in DEPC treated water) using a SYBR green dye in a LightCycler 480 II (Roche Diagnostics, Laval, QC, Canada) as previously described. Specific sequences of primers were described previously [29,30,31].

### 2.5. Stimulation of Chicken Macrophages with Tracheal Supernatants

A chicken macrophage cell line (MQ-NCSU), was kindly provided by Dr. Juan Carlos Rodriguez (University of Prince Edward Island, Charlottetown, PE, Canada). The cells were seeded into 48-well cell culture plates at a viable cell density (determined by trypan blue exclusion) of 5 × 10^5^ cells/well in Dulbecco's Modified Eagle's medium (DMEM) (Gibco) containing 10% fetal bovine serum (FBS), 200 U/mL penicillin, and 80 µg/mL streptomycin for two hours. Cells were then treated for 48 h with supernatants from cultured tracheal rings. Macrophage culture supernatants were collected to quantify nitric oxide (NO) production by Griess assay (Promega, Madison, WI, USA) according to the manufacturer’s instructions.

To determine the antiviral activities of tracheal ring supernatants, chicken macrophages were treated with supernatants for 6 h prior to AIV infection. Cells were infected with H4N6 AIV as described previously [23]. Briefly, MQ-NCSU cells were seeded at 5 × 10^5^ cells/mL into 24-well cell culture plates in (DMEM) supplemented with 10% heat-inactivated FBS, 200 U/mL penicillin, and 80 µg/mL streptomycin and 50 µg/mL gentamicin for two hours incubation at 40 °C. Cells were then treated with tracheal ring supernatants for 6 h. Cells were infected with H4N6 AIV at a multiplicity of infection (MOI) of 1.0; residual virus was removed two hours post-infection by washing twice with warm medium. The virus titer in macrophage supernatants was measured using a 50% tissue culture infective dose (TCID_50_) assay at 12 h post-infection.

### 2.6. Statistical Analysis

Statistical analysis of log transformed virus titers was performed by one-way ANOVA (SAS version 9.3) followed by Tukey’s post hoc test for multiple comparisons to examine the effects of TLR ligands. *p* values <0.05 were considered to be statistically significant. For gene expression, fold changes and standard errors were calculated using REST (Relative Expression Software Tool) software version 2009 (Qiagen, Toronto, ON, Canada). The REST software compared control (medium or non CpG ODN) and treatment groups using pair-wise fixed reallocation randomization based on the PCR efficiencies and the mean crossing point deviations between the control and treatment groups. Statistical analysis of Nitric oxide production was performed by two-tailed Student’s *t*-test.

## 3. Results

### 3.1. TLR Ligands Induce the Expression of Innate Response Genes in Chicken Tracheal Organ Cultures

**Figure 1:**
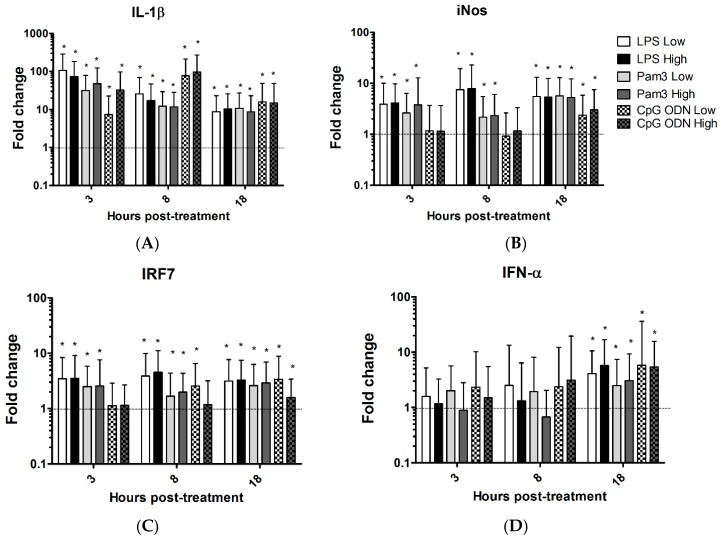

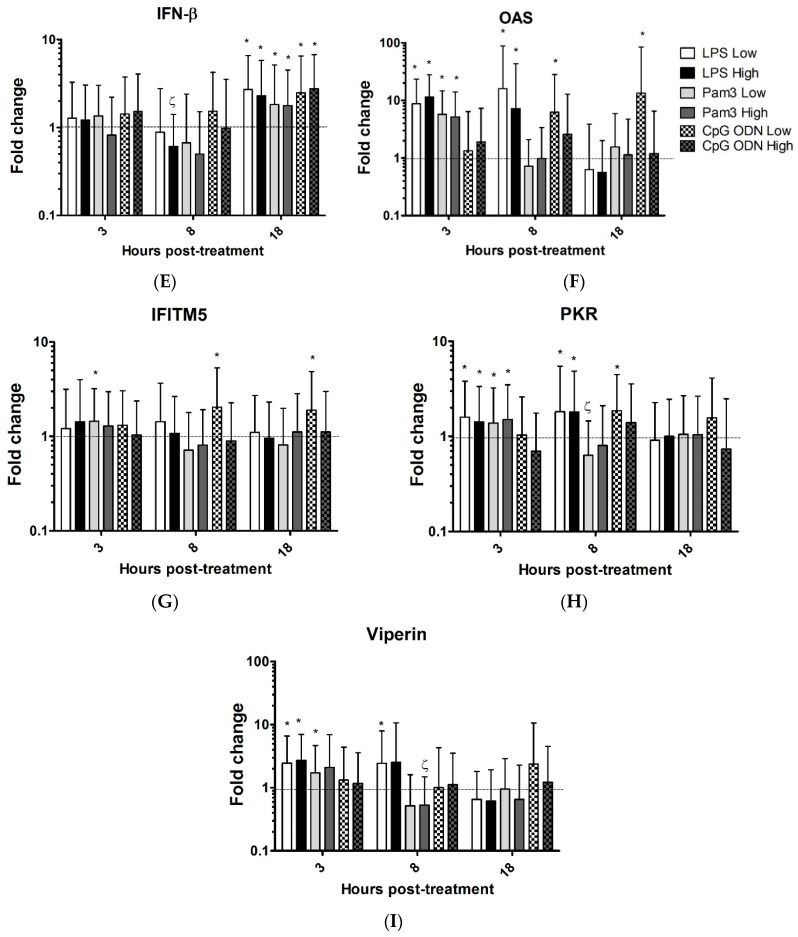
Relative expression of candidate genes interleukin 1 beta (IL-1β) (**A**); inducible nitric oxide synthase (iNOS) (**B**); interfon (IFN) regulatory factor 7 (IRF7) (**C**); IFN-α (**D**); IFN-β (**E**); oligoadenylate synthetase (OAS) (**F**); IFN induced transmembrane protein 5 (IFITM5) (**G**); protein kinase R (PKR) (**H**); Viperin (I), in the chicken trachea. Tracheal organ cultures (TOCs) were treated with two different concentrations of lipopolysaccharide (LPS) from *Escherichia coli* (*E. coli*) O26:B6 (1.0 µg/mL (LPS low) and 10 µg/mL (LPS high)), Pam3CSK4 (1.0 µg/mL (Pam3 low) and 10 µg/mL (Pam3 high)), CpG ODN 1826 (1.0 µg/mL (CpG ODN low) and 10 µg/mL (CpG ODN high)). Control groups received either non CpG ODN (10 µg/mL) or medium. Gene expression was assessed at 3, 8 and 18 h post-treatment using quantitative real-time (RT)-PCR, relative to the housekeeping gene β-actin. Gene expression is presented as fold change relative to the medium group in LPS and Pam3CSK4 (low and high doses) groups. Gene expression is presented as fold change relative to the non CpG ODN group in CpG (low and high doses) groups. Error bars represent standard errors of the means. Fold changes and standard errors were calculated using REST software. Significant up-regulation (*p* ≤ 0.05) is indicated by *. Significant down-regulation is indicated by ζ. There were six biological replicates in each group.

The expression of interleukin 1 beta (IL-1β) was significantly increased in the TOCs treated with Pam3CSK4, LPS and CpG ODN compared to untreated rings at all time points (Figure 1A, Table 1). Treatment of TOCs with low dose or high dose of Pam3CSK4 or LPS significantly up-regulated the expression of inducible nitric oxide synthase (iNOS) at 3, 8, and 18 h post-treatment. However, the expression of iNOS by TOCs that received low dose and high dose of CpG ODN was significantly increased only at 18 h post-treatment (Figure 1B, Table 1).

Low dose and high dose of Pam3CSK4 or LPS significantly up-regulated interferon regulatory factor (IRF)7 at 3, 8 and 18 h post-treatment. Low dose and high dose of CPG ODN significantly up-regulated IRF7 at 18 h post-treatment. In addition, the low dose of CpG ODN significantly up-regulated the expression of IRF7 at 8 h post-treatment (Figure 1C, Table 1). Treatment of TOCs with Pam3CSK4, LPS and CpG ODN significantly induced the expression of IFN-α at 18 h post-treatment compared to untreated TOCs (Figure 1D, Table 1). The expression of IFN-β by TOCs incubated with low or high doses of LPS or CpG ODN was significantly up-regulated at 18 h post-treatment. However, high dose of LPS significantly down-regulated the expression of IFN-β in TOCs at 8 h post-treatment (Figure 1E, Table 1). Low or high dose of Pam3CSK4 significantly induced the expression of OAS by TOCs at 3 h post-treatment whereas low dose or high dose of LPS significantly up-regulated the expression of OAS at 3 and 8 h post-treatment. The expression of OAS by TOCs that received low dose of CpG ODN was significantly increased at 8 and 18 h post-treatment (Figure 1F, Table 1). In TOCs treated with low dose of Pam3CSK4 or CpG ODN, the expression of IFITM5 was significantly increased at 8 h post-treatment. The expression of IFITM5 by TOCs that received low dose of CpG ODN was significantly increased at 18 h post-treatment (Figure 1G, Table 1). Treatment of TOCs with low dose or high dose of Pam3CSK4 caused significant up-regulation of PKR at 3 h post-treatment, while the expression of this gene was up-regulated at 8 h post-treatment in the group treated with low dose of CpG ODN or LPS. In addition, the high dose of LPS significantly induced the up-regulation of PKR at 3 h post-treatment (Figure 1H, Table 1).

Low dose or high dose of LPS caused significant up-regulation of viperin at 3 h post-treatment. In addition, the expression of viperin by TOCs that received low dose of Pam3CSK4 was significantly increased at 3 h post-treatment. Low dose of LPS significantly induced the up-regulation of viperin at 8 h post-treatment. However, high dose of Pam3CSK4 down-regulated the expression of viperin at 8 h post-treatment (Figure 1I, Table 1).

**Figure 2:**
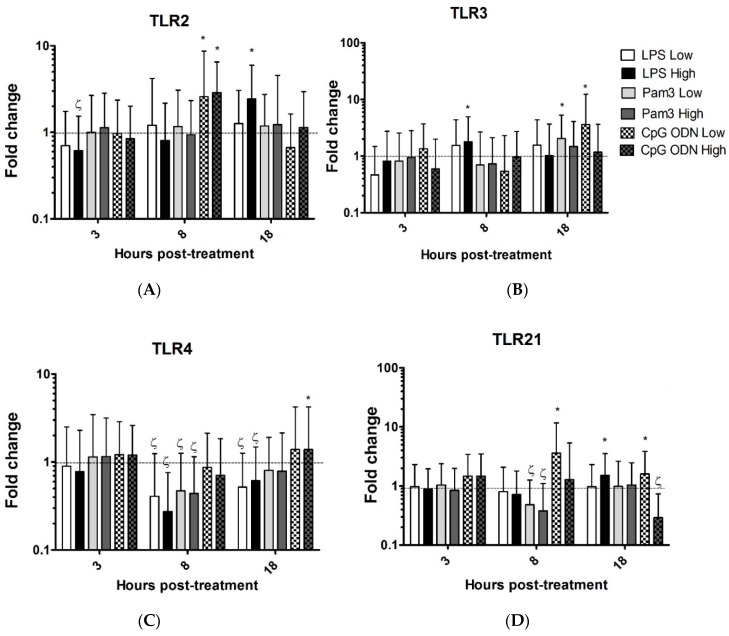
Relative expression of Toll-like receptor (TLR) 2 (**A**), 3 (**B**), 4 (**C**) and 21 (**D**) in the trachea. TOCs were treated with two concentrations of LPS from *E. coli* O26:B6 (1.0 µg/mL (LPS low) and 10 µg/mL (LPS high)), Pam3CSK4 (1.0 µg/mL (Pam3 low) and 10 µg/mL (Pam3 high)), CpG ODN 1826 (1.0 µg/mL (CpG ODN low) and 10 µg/mL (CpG ODN high)). Control groups received either non CpG ODN (10 µg/mL) or medium. Gene expression was assessed at 3, 8 and 18 h post-treatment using quantitative RT-PCR, relative to the housekeeping gene β-actin. Gene expression is presented as fold change relative to the medium group in LPS and Pam3CSK4 (low and high doses) groups. Gene expression is presented as fold change relative to the non CpG ODN group in CpG (low and high doses) groups. Error bars represent standard errors of the means. Fold changes and standard errors were calculated using REST software. Significant up-regulation (*p* ≤ 0.05) is indicated by *. Significant down-regulation is indicated by ζ. There were six biological replicates in each group.

High and low dose of CpG ODN significantly induced the expression of TLR2 at 8 h post- treatment. High dose of LPS significantly induced the expression of TLR2 at 18 h post-treatment, however, the high dose of LPS significantly down-regulated the expression of TLR2, at 3 h post-treatment (Figure 2A, Table 1). High dose of LPS significantly induced the up-regulation of TLR3 at 8 h post-treatment. Low dose of Pam3CSK4 and CpG ODN significantly induced the expression of TLR3 at 18 h post-treatment (Figure 2B, Table 1). The expression of TLR4 by TOCs treated with either Pam3CSK4 or LPS was significantly down-regulated, however, its expression was significantly increased by high dose of CpG ODN at 18 h post-treatment (Figure 2C, Table 1). The expression of TLR21 by TOCs treated with low dose or high dose of Pam3CSK4 was significantly down-regulated at 8 h post-treatment, while low dose of CpG ODN significantly induced the expression of TLR21 at 8 h post-treatment. In addition, high dose of LPS and low dose of CpG ODN significantly induced the expression of TLR21 at 18 h post-treatment. However, the expression of TLR21 by TOCs treated with high dose of CpG ODN was significantly down-regulated at 18 h post-treatment (Figure 2D, Table 1). TLR ligands did not significantly affect the expression of TLR7 (data not shown).

### 3.2. Supernatants from Chicken Tracheas Treated with TLR Ligands Activate Chicken Macrophages

**Figure 3:**
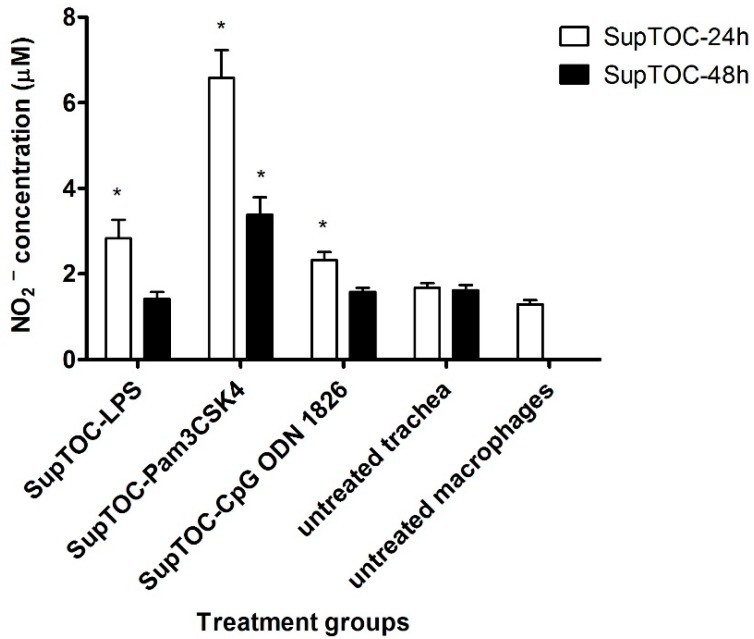
Nitrite oxide (NO_2_^−^) production in chicken macrophage cells (MQ-NCSU cell line). Chicken macrophages were stimulated with supernatants from TOCs (that were previously stimulated with TLR ligands). Nitrite oxide in MQ-NCSU supernatants was measured after 48 h of stimulation, via the Griess assay. Nitric oxide production in each group was compared to the cells without stimulation using a two-tailed Student’s *t*-test. Significant differences (*p* ≤ 0.05) between a test group and the group without receiving supernatant from TOC are indicated by *. SupTOC-24: culture supernatants from TOCs collected 24 h post-treatment with TLR ligands or medium. SupTOC-48: culture supernatants from TOCs collected 48 h post-treatment with TLR ligands or medium.

To determine whether culture supernatants of treated tracheal rings were able to activate chicken macrophages, MQ-NCSU cells were treated for 48 h with culture supernatants from treated tracheal rings. Culture supernatants of tracheal rings collected 24 h post-treatment with LPS (SupTOC-LPS-24h), SupTOC-PamCSK4-24 and SupTOC-CpG-24h significantly induced nitrite production by MQ-NCSU cells. In addition, SupTOC-PamCSK4-48 significantly induced nitrite production by chicken macrophages compared to untreated chicken macrophages, however, SupTOC-LPS-48h, and SupTOC-CpG-48h did not induce nitrite production by these cells (Figure 3).

### 3.3. Antiviral Compounds Produced by Stimulated TOCs Are Able to Limit AIV Replication in Chicken Macrophages

**Figure 4:**
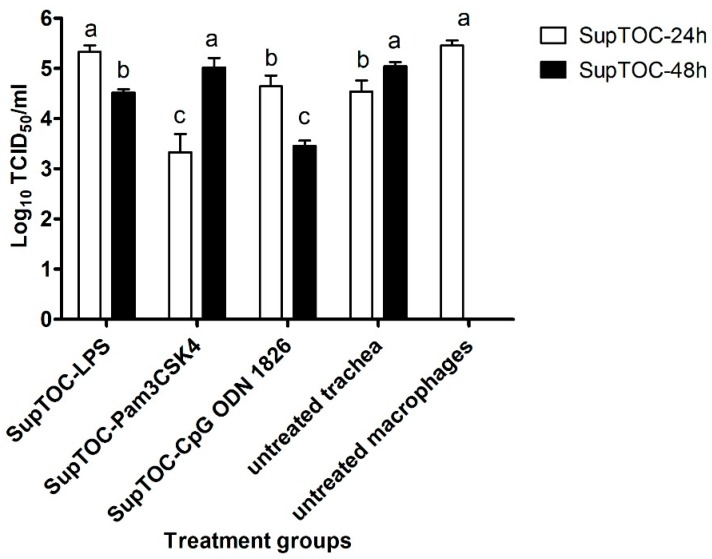
Supernatants from TOCs stimulated with TLR ligands reduced influenza virus replication in chicken macrophage cells. There were five groups. Three of them were treated with supernatants from TOCs treated with LPS from *E. coli* O26:B6, Pam3CSK4 and CpG ODN 1826 and one was treated with supernatant from TOCs that received only medium (untreated trachea), and the last group did not receive supernatant from TOCs. Cells were infected 6 h later with H4N6 avian influenza virus (MOI of 1.0). Virus titer (12 h after infection) was quantified by means of a TCID_50_ assay. a–c columns with no common letter are significantly different (*p* ≤ 0.05). SupTOC-24h: culture supernatants from TOCs collected 24 h post-treatment with TLR ligands or medium. SupTOC-48h: culture supernatants from TOCs collected 48 h post-treatment with TLR ligands or medium.

Culture supernatants from TOCs treated with TLR ligands significantly decreased viral replication in chicken macrophages, confirming antiviral activity against AIV (Figure 4). Treating chicken macrophages with SupTOC-LPS-48h significantly reduced virus titers by 8.5-fold in their supernatants compared to the infected, untreated macrophages. In contrast, treatment of chicken macrophages with SupTOC-LPS-24h did not significantly reduce virus titer in macrophage supernatants (Figure 4).

Macrophages treated with SupTOC-Pam3CSK4-24h significantly reduced virus titers by 131-fold in their supernatants compared to the infected, untreated macrophages. However, treatment of chicken macrophages with SupTOC-Pam3CSK4-48h did not significantly reduce virus titers in macrophage supernatants compared to untreated, infected macrophages (Figure 4).

Treating chicken macrophages with SupTOC-CpG-24h significantly reduced virus titers by 6.5-fold in their supernatants compared to the infected, untreated macrophages while the treatment of chicken macrophages with SupTOC-CpG-48h significantly reduced virus titers by 100 fold in their supernatants compared to the infected, untreated macrophages. Moreover, treatment of chicken macrophages with SupTOC-CpG-48h significantly reduced virus replication in chicken macrophages compared to treatment of chicken macrophages with SupTOC-CpG-24h (Figure 4).

## 4. Discussion

Tracheal organ culture has been used for culturing viruses, such as avian infectious bronchitis virus [33] as well as examining virus pathogenesis [34,35]. This method has the advantage of allowing to determine local innate responses within the trachea. TOC allows interaction to occur among cell types, which cannot be modeled using cell lines or isolated primary cells. The TOC described here enabled us to determine innate antiviral responses of chicken tracheas treated with TLR ligands.

This study extended the findings of a previous study in which chickens were intranasally treated with TLR ligands and demonstrated significant up-regulation of candidate genes involved in antiviral responses in the trachea [29]. The current study made it possible to determine innate responses induced by TLR ligands in the trachea once the trachea was locally stimulated with TLR ligands. All TLR ligands examined in this study induced the expression of IL-1β in TOC. The induced IL-1β in TOCs following TLR ligand treatment could be a trigger for NO production in macrophages, as it has been shown that IL-1β can initiate NO synthesis [36]. Furthermore, IL-1β induces the proliferation of T and B cells and recruit lymphocytes to the trachea. It is possible that this cytokine recruits and activates macrophages and heterophils in the respiratory tract to inhibit the spread of infection in the host. Therefore, induction of IL-1β in the trachea may play a role in antiviral responses against AIV through its chemotactic effect. In addition, we confirmed functional activity of supernatants of TOC treated with TLR ligands. To this end, we treated macrophages with supernatants from TOCs treated with TLR ligands. We showed that supernatants from TOC treated with TLR2, 4 and 21 ligands activated chicken macrophages as indicated by NO production in macrophages. NO production is a reliable method to determine macrophage activation [37]. The same scenario may apply to in vivo conditions whereby macrophages play important roles in host defense against infection of the trachea by viral pathogens [38].

Antiviral responses are necessary to suppress viral replication and virus spread within the host. TLR ligands including LPS, Pam3CSK4 and CpG ODN induce antiviral responses in chickens against AIV which leads to reduction of viral shedding in infected chickens [29]. In the current study, the induction of antiviral responses following TLR ligand treatment was demonstrated by induced expression of IRF7 and ISGs in TOCs and the reduction of AIV replication in chicken macrophages treated with TOC supernatants. Engagement of TLRs with their ligands results in triggering several intracellular pathways leading to the expression of cytokines and other antiviral mechanisms. This process is mediated by transcription factors, such as IRF7 [39]. In agreement with previous studies [30,31], all three TLR ligands tested in this study induced the expression of IRF7, an important transcription factor in TLR signaling pathways and downstream responses [40]. In the present study, all three TLR ligands induced the expression of type I IFNs in TOCs. The critical role of type I IFNs in innate responses against influenza virus has been well documented in mammalian species and chickens. In humans, it has been demonstrated that the lack of IFN-β induction results in impaired responses against influenza virus [41,42]. In addition, the importance of type I IFNs in the induction of ISGs, which play a critical role in antiviral responses against influenza virus, has been well established [18,23,24,43,44,45]. However, we observed that TLR ligands induced the early expression of OAS, viperin and PKR in TOCs even prior to the induction of type I IFNs. Significant up-regulation of OAS, viperin and PKR prior to the expression of type I IFNs in TOCs may support IFN-independent activation of these ISGs, an observation that has been previously reported in humans and mice [46,47]. Moreover, we determined the expression of IFITM5 in TOCs following CpG ODN and Pam3CSK4 treatment. Members of the IFITM family, which are also classified as ISGs, have primary antiviral functions in host cells. IFITM proteins prevent the fusion of influenza virus to cell membrane and therefore inhibit the entry of influenza virus into host cells [25,26,48].

In addition to the induction of intracellular signaling pathways by TLR ligands, they induce the expression of TLRs in host cells to enhance the ability of the host to recognize the virus [17,49,50]. In the current study, CpG ODN and Pam3CSK4 induced the expression of TLR3 in TOCs. TLR3 recognizes the presence of viral RNA in infected cells. The function of TLR3 and its importance in the induction of antiviral responses against influenza virus in mammals and chickens have been previously emphasized [17,51].

In the present study, antiviral activity of TOCs treated with TLR ligands was investigated. Subsequent to treatment of chicken macrophages with supernatants of TOCs treated with TLR ligands, AIV replication was significantly reduced in macrophages. This antiviral activity suggested that supernatants from TOCs may contain IFNs and ISGs or other active antiviral components. Importantly, our findings underlined the differences among TLR ligands for induction of ISGs in TOCs, which may correlate with their ability to reduce viral replication. For example, the inability of LPS to induce significant expression of IFITM5 and TLR3 in TOCs may explain the limited reduction of AIV replication in chicken macrophages following treatment with SupTOC-LPS compared to supernatants from TOCs treated with Pam3CSK4 or CpG.

The results presented here also highlighted the influence of incubation time following TLR ligand treatment on the ability of TOC supernatants to limit AIV replication in chicken macrophages. SupTOC-Pam3CSK4-24h, SupTOC-LPS-48h and SupTOC-CpG-48h significantly reduced AIV replication in chicken macrophages. The difference in the effects of incubation time between TOCs treated with different TLR ligands might be correlated with the nature and quantity of the antiviral compounds produced by TOCs during the incubation. This interpretation is supported by a previous study which showed that LPS and Pam3CSK4 induced varying profiles of genes involved in the regulation of type I IFNs [52]. The activation and regulation of type I IFN signaling pathway could affect trachea responses which was reflected in TOC supernatants with different incubation time. Along with regulatory mechanisms of type I IFN signaling pathways, the kinetics and the amount of IFN produced by host cells depend on the type and the complexity of TLR ligands [53]. The delineated differences of TOC capability to induce antiviral responses is of importance and further studies are required to detect and quantify components in TOC supernatants.

In the present study, untreated TOCs showed basal expression of type I IFNs and ISGs, and antiviral activities against AIV. Tracheal cells, especially epithelial cells, are able to induce innate responses, however, the responses mounted by trachea in vivo may not be sufficient to overcome a viral infection.

## 5. Conclusions

In conclusion, we have demonstrated that chicken tracheal organ cultures are able to mount antiviral responses after stimulation with different TLR ligands. This was confirmed by examining gene expression in tracheal organ cultures. Supernatants harvested from organ cultures treated with TLR ligands had the capacity to activate macrophages and limit AIV replication in these cells. Future studies are required to evaluate the relative importance of the molecules, especially the ISGs, which we have measured in the present study.

## Figures and Tables

**Table 1:** Cytokine gene expression of chicken TOC stimulated with Pam3CSK4 (1.0 µg/mL (Pam3 low) and 10 µg/mL (Pam3 high)), LPS from *E. coli* O26:B6 (1.0 µg/mL (LPS low) and 10 µg/mL (LPS high)), and class B CpG ODN 1826 (1.0 µg/mL (CpG ODN low) and 10 µg/mL (CpG ODN high)).

**Genes**		**LPS Low**				**LPS Highv**				**Pam Low**				**Pam High**				**CpG ODN LOW**				**CpG ODN High**		
		**3 h**	**8 h**	**18 h**		**3 h**	**8 h**	**18 h**		**3 h**	**8 h**	**18 h**		**3 h**	**8 h**	**18 h**		**3 h**	**8 h**	**18 h**		**3 h**	**8 h**	**18 h**
IL-1B		107↑	26↑	8.7↑		73↑	17↑	10↑		32↑	12↑	11↑		48↑	12↑	8.7↑		7.3↑	77↑	16 ↑		33↑	97↑	15↑
iNOS		3.9↑	7.5↑	5.5↑		4.1↑	7.9↑	5.4↑		2.6↑	2.2↑	5.7↑		3.8↑	2.3↑	5.3↑				2.4↑				3↑
IRF7		3.5↑	3.9↑	3.2↑		3.5↑	4.5↑	3.3↑		2.5↑	1.7↑	2.6↑		2.6↑	2↑	2.9↑			2.6↑	3.4↑				1.6↑
IFN-α				4 ↑				5.7↑			1.9↑	2.5↑				3.0↑				5.8↑				5.4↑
IFN-β				2.7↑			1.7↓	2.3↑								1.8↑				2.5↑				2.8↑
OAS		8.7↑	16↑			12↑	7.3↑			5.7↑				5.2↑					6.3↑	13.4↑				
IFITM5										1.5↑									1.9↑	2↑				
PKR		1.6↑	1.8↑			1.4↑	1.8↑			1.4↑	1.7↓			1.5↑					1.9↑					
viperin		2.5↑	2.5↑			2.7↑				1.7↑					2↓									
TLR2						1.7↓		2.4↑											2.6↑				2.9↑	
TLR3							1.8↑					2.0↑								3.6↑				
TLR4			2.5↓	2↓			3.3↓	1.7↓			2.0↓				2.5↓									1.3↑
TLR21								1.5↑			2↓				2.5↓				3.5↑	1.6↑				3.3↓

Gene expression is presented as fold change relative to either non CpG ODN group or medium groups. Significant up-regulation (*p* ≤ 0.05) is indicated by ↑. Significant down-regulation (*p* ≤ 0.05) is indicated ↓. LPS: lipopolysaccharide; Pam: Pam3CSK4; IL-1β: interleukin 1 beta; iNOS: inducible nitric oxide synthase; IRF7: interferon (IFN) regulatory factor 7; OAS: oligoadenylate synthetase; IFITM5: IFN induced transmembrane protein 5; PKR: protein kinase R; TLR: Toll-like receptor.
